# Nanozyme-Driven Multiplex Signal Lateral Flow Immunoassays for Chemical Contaminants in Food: A Review

**DOI:** 10.3390/bios16060342

**Published:** 2026-06-17

**Authors:** Jiaqi Chen, Xingtian Wei, Yihao Shi, Yang Piao, Jiakang He, Hailan Chen, Jincheng Xiong, Lilan Lyu, Liang Luo

**Affiliations:** 1 College of Animal Science and Technology, Guangxi Key Laboratory of Animal Breeding, Disease Control and Prevention, Guangxi University, Nanning 530004, China; 2 Guangxi Subtropical Crops Research Institute, Nanning 530001, China; 3 Institute of Quality Standard and Monitoring Technology for Agro-Products of Guangdong Academy of Agricultural Sciences, Guangzhou 510640, China

**Keywords:** nanozyme, multiplex signal readout, lateral flow immunoassay, chemical contaminant, food safety

## Abstract

Chemical contaminants in food pose a serious threat to public health, driving the need for sensitive, rapid, and *on-site* screening methods. Lateral flow immunoassay (LFIA) is rapid and portable but suffers from single-signal readout and insufficient label stability. Nanozymes, nanomaterials with enzyme-like catalytic activity and excellent stability, have emerged as promising signal labels to address these limitations. Moreover, their diverse physiochemical properties enable multiplex signal readout, where two or more complementary signals (e.g., colorimetric, fluorescent, chemiluminescent, photothermal, and surface-enhanced Raman scattering) are generated simultaneously from a single test line. This multiplex strategy significantly enhances detection sensitivity, accuracy, and reliability through signal amplification and self-calibration. This review provides a systematic overview of the catalytic properties and their major types used in multiplex signal LFIA. The signal combination strategies employed in nanozyme-based multiplex signal LFIA were also summarized, and their applications in detecting veterinary drugs, mycotoxins, pesticides, and other food chemical contaminants are highlighted. Ultimately, current challenges and future prospectives in this field are discussed. This review offers guidance for designing high-performance, nanozyme-based multiplex signal LFIA platforms for food safety monitoring.

## 1. Introduction

Chemical contaminants, including veterinary drugs, mycotoxins, pesticides, illegal food additives, and heavy metal ions, may be present at trace levels in food throughout the entire chain of production, processing, transportation, and storage [1,2,3,4]. Long-term exposure to these contaminants is associated with antimicrobial resistance, carcinogenicity, endocrine disruption, neurodevelopmental disorders, acute poisoning, and even death, posing substantial threats to public health [5,6,7,8]. Therefore, sensitive, accurate, rapid, and *on-site* screening methods are urgently needed for effective good safety regulation.

Conventional instrumental methods such as high-performance liquid chromatography (HPLC), high-performance liquid chromatography–mass spectrometry (HPLC-MS), gas chromatography (GC), and gas chromatography–mass spectrometry (GC-MS) offer excellent sensitivity and specificity [9,10,11]. However, these techniques usually require costly instruments, complex sample pretreatment, and trained personnel, making them unsuitable for large-scale *on-site* monitoring.

Immunoassays, particularly lateral flow immunoassay (LFIA), have emerged as powerful alternatives due to their user-friendly operation, rapidity, and cost-effectiveness [12,13,14]. Nevertheless, the performance of conventional LFIA is constrained by two principal limitations. First, conventional LFIA depends on the aggregation of colloidal gold nanoparticle (GNP) labels that possess a low molar extinction coefficient and limited stability to generate a colorimetric signal, resulting in compromised sensitivity and accuracy [15]. Moreover, GNP labels provide only a single colorimetric signal suitable for visual qualitative readout and are incapable of delivering the quantitative data required for precise determination of trace-level food contaminants [16]. Recently, multiplex signal readout strategies that involve two or more complementary output signals for quantification have been integrated into LFIA systems. These approaches utilize a colorimetric signal for rapid qualitative screening, while another signal, such as fluorescence, chemiluminescence, photothermal, electrochemical, or surface-enhanced Raman spectroscopy (SERS), enables accurate target quantification [17,18,19]. Compared with single-signal readout, multiplex signal readout offers distinct advantages: independent signal channels enable self-calibration and self-verification against non-target interferences, significantly enhancing both sensitivity and accuracy; moreover, a suitable output signal can be selected according to specific scenarios, improving detection flexibility [20]. The key to realizing such multiplex signal LFIAs lies in the rational design of signal labels capable of simultaneous multiple signal output.

Nanozymes, a class of nanomaterials with enzyme-like activity, enable catalysis of natural enzyme substrates and related biochemical reactions [21]. Unlike conventional labels, nanozymes not only exhibit intrinsic physicochemical properties (e.g., color, fluorescence, magnetism, or photothermal conversion) but also possess catalytic activities (e.g., peroxidase (POD)-like or oxidase (OXD)-like) that can amplify signals or generate new ones [22,23,24,25,26]. For example, Fe_3_O_4_ nanozymes combine magnetic properties with POD-like activity, enabling both target enrichment and signal readout [27,28]; perovskite nanozymes exhibit strong fluorescence emission and POD-like activity, allowing simultaneous fluorescent and catalytic colorimetric signals [29,30]; and gold nanozymes integrate SERS activity with catalytic capability, enabling dual-mode detection [31,32]. These multifunctional properties make nanozymes particularly attractive for developing multiplex signal LFIA platforms.

Given the serious risks posed by chemical contaminants in food and the critical role of LFIA as a rapid *on-site* screening tool, a comprehensive summary and critical evaluation of the progress of nanozyme-driven multiplex signal LFIAs in detecting these contaminants is of great significance. Recently, several reviews have summarized nanozyme-based biosensing in the food field. Sofiene Mansouri et al. [33] reviewed nanozyme-based dual- and multiple-mode biosensing for food safety and environmental applications, but their review did not focus on LFIAs. Moreover, this group also provided an overview of nanozyme-based LFIAs, yet this work primarily concentrated on pathogenic microorganisms and toxins in food, with limited discussion of multiple signal readout strategies [34]. Shen et al. [35] summarized advances in the application of nanozymes for detection of contaminants in animal-derived foods. However, their discussion was limited to veterinary drug residues and did not specifically focus on LFIAs either. Overall, these reviews provide valuable insights into nanozyme-based LFIAs in food safety. However, there is still a lack of a systematic review of nanozyme-based multiplex signal LFIA for food chemical contaminants, especially in terms of signal combination strategies and practical applications.

**Scheme 1:**
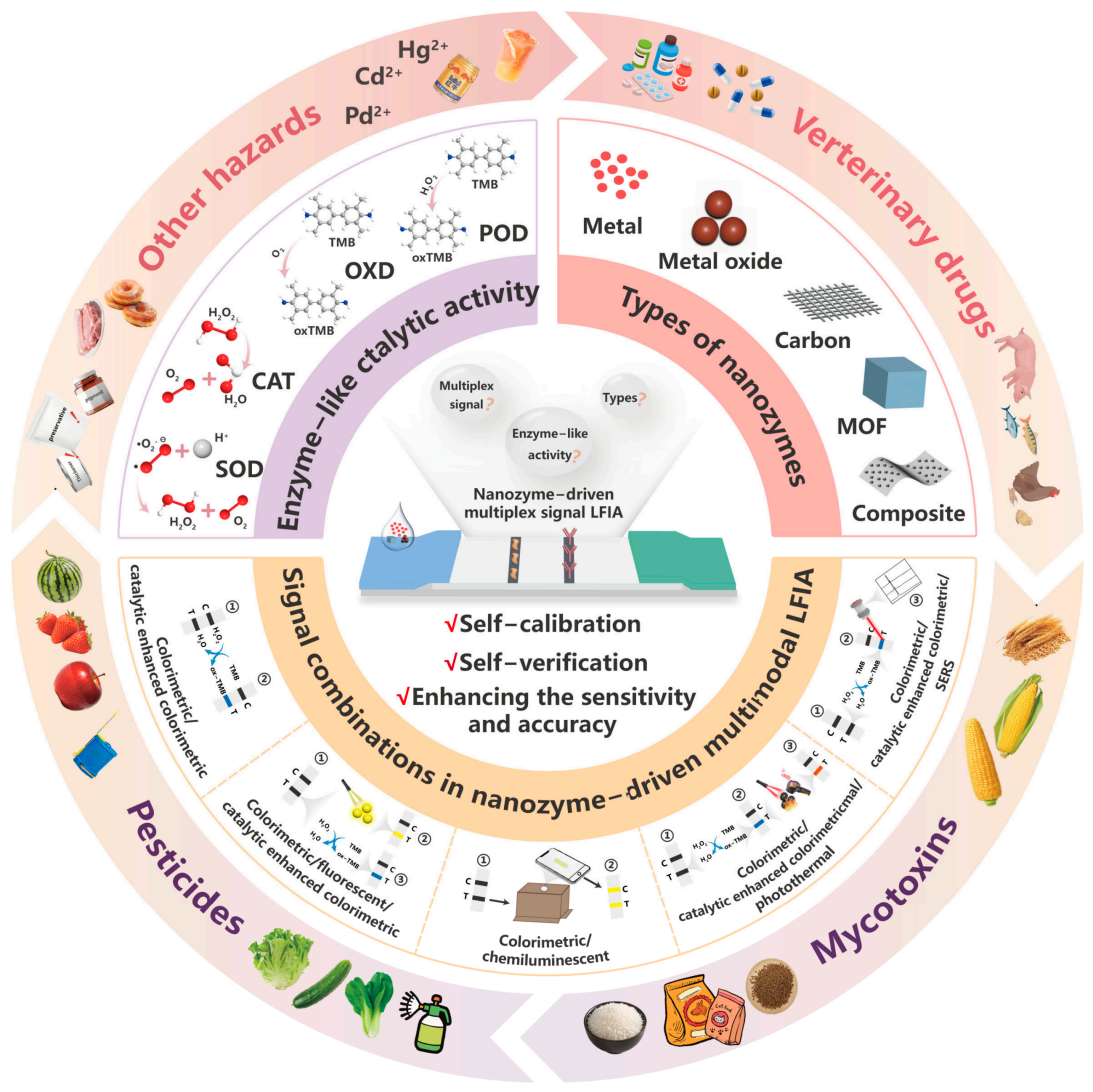
Overview of nanozyme-driven multiplex signal LFIA and its applications in food chemical contaminants detection.

To address this gap, this review presents the first comprehensive and critical overview of the advances in nanozyme-based multiplex signal LFIAs for detecting chemical contaminants in food over the last seven years. We first introduce the catalytic properties and classifications of nanozymes used in multiplex LFIAs. Subsequently, we summarize the signal combination strategies employed in these platforms and highlight their applications for detecting veterinary drugs, mycotoxins, pesticides, and other hazardous substances. Ultimately, we critically discuss current challenges and future perspectives, including improving catalytic efficiency, integrating machine learning for data interpretation, developing portable readout devices, and achieving simultaneous multi-analyte detection (Scheme 1).

## 2. Catalytic Properties and Types of Nanozymes in Multiplex Signal LFIA

Due to the inherent enzyme-like catalytic activity of nanozymes and their ability to support multiple signal output characteristics (e.g., color, fluorescence, photothermal conversion), they have been widely used as functional labels in multiplex signal LFIA platforms. Therefore, understanding their catalytic properties and classifications is fundamental to designing high-performance LFIA platforms. This section first introduces the major types of enzyme-like catalytic activities, then categorizes the nanozymes commonly employed in multiplex signal LFIAs based on their material composition.

### 2.1. Enzyme-like Catalytic Properties of Nanozyme

**Figure 1:**
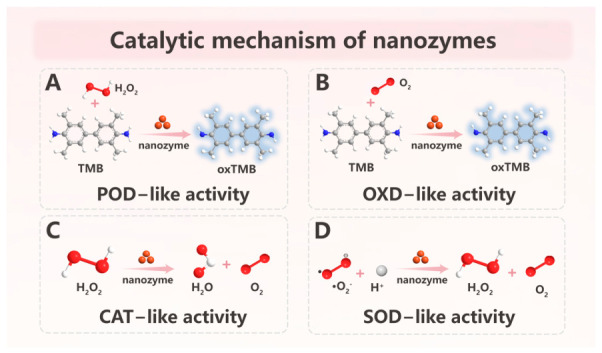
Catalytic mechanism of nanozymes. (**A**) POD-like, (**B**) OXD-like, (**C**) CAT-like, (**D**) SOD-like activities.

As nanomaterials with enzyme-like activity have been widely used in various types of sensors, nanozymes have been found to possess diverse types of catalytic activities, primarily including POD-like, OXD-like, catalase (CAT)-like, and superoxide dismutase (SOD)-like activities [36,37,38]. The specific catalytic mechanisms of these nanozyme activities are illustrated in Figure 1. Among them, POD-like activity is both the earliest discovered and the most extensively reported. This catalytic activity primarily proceeds via the generation of reactive oxygen species (ROS) or electron transfer processes. Specifically, POD-like nanozymes catalyze H_2_O_2_ to generate ROS such as hydroxyl radicals (•OH), which then oxidize specific substrates (e.g., converting chromogenic substrate 3,3′,5,5′-tetramethylbenzidine (TMB) into oxidized TMB) (Figure 1A). In electron transfer processes, nanozymes act as electron transfer mediators, accelerating redox reactions by facilitating electron transfer between the substrate and H_2_O_2_. Generally, the POD-like nanozymes follow typical Michaelis–Menten kinetics and a lower K_m_ value indicates higher substrate affinity [39]. Ji et al. [40] compared FeN_3_P-SAzyme and horseradish peroxidase (HRP) through kinetic analysis. The K_m_ for FeN_3_P-SAzyme was 2.06 × 10^−3^ mM, while that of HRP was 5.55 mM. Panferov et al. [41] compared the kinetic parameters of the POD-mimicking Au@Pt nanozymes with HRP. They found that the K_m_ of Au@Pt for TMB was 0.21 mM, which is comparable to that of HRP (0.38 mM). However, the K_m_ of Au@Pt for H_2_O_2_ was three orders of magnitude higher than that of HRP, indicating that POD-like nanozymes have high affinity for TMB substrate but a lower affinity for H_2_O_2_. To date, a wide variety of nanozymes, such as metal nanozymes, metal oxide nanozymes, carbon nanozymes, and metal organic framework (MOF) nanozymes, have been demonstrated to exhibit POD-like activity [42]. For example, Zhang et al. [43] developed a Ru nanoparticle-modified TiO_2_ (Ru@TiO_2_) POD-like nanozyme. The K_m_s of this nanozyme for TMB and H_2_O_2_ were 0.20 × 10^−3^ M and 0.51 × 10^−3^ M, respectively, indicating a high affinity for both. Based on its excellent catalytic performance, colorimetric detection of miRNAs was achieved. Liu et al. [44] designed a bimetallic Co/Mn-MOFs with high POD-like activity, exhibiting a K_m_ of 3.94 mM and a maximum reaction velocity (V_max_) of 2.41 × 10^−8^ M/s toward the chromogenic substrate TMB. Based on this Co/Mn-MOFs POD-like nanozyme, a simple and highly sensitive colorimetric sensing platform was successfully proposed for chlorpyrifos detection.

Compared with POD-like activity, OXD-like nanozymes catalyze the oxidation of substrates using O_2_ as the electron acceptor, yielding H_2_O_2_ or H_2_O through a pathway that similarly involves electron transfer and the generation of reactive species (Figure 1B). Similarly, OXD-like nanozymes also follow typical Michaelis–Menten kinetics. Liang et al. [45] reported that Mn_3_O_4_ nanozymes exhibited a lower K_m_ toward TMB (0.1896 mM) than HRP (0.434 mM), indicating that OXD-like nanozymes exhibit strong substrate affinity. Overall, compared with the natural enzyme HRP, both POD-like and OXD-like nanozymes show comparable or lower K_m_ values for substrates. This may be attributed to their higher specific surface area and greater number of active sites, which improve substrate adsorption and binding for substrate affinity enhancement [46]. In addition, the number of reported OXD-like nanozymes is relatively limited compared to that of POD-like nanozymes, and these OXD-like nanozymes are primarily based on transition metal elements such as Ce, Co, Cu, Mn, Fe, and Ni [42]. Because they lack the need for unstable H_2_O_2_ for substrate oxidation, OXD-like nanozymes have attracted considerable interest for analytical applications. Lv et al. [47] synthesized MnO_2_ nanoflowers and found that aptamer adsorption markedly enhanced their OXD-like activity in a sequence length-dependent manner. Based on this observation, a facile label-free colorimetric aptasensor for ochratoxin A detection was established. Luo et al. [48] also synthesized MnO_2_ nanosheets with excellent OXD-like activity and proposed a ratiometric fluorescence sensing platform for ractopamine (RAC) detection based on a nanozyme–enzyme cascade reaction. In this system, MnO_2_ nanosheets catalyze the oxidation of Amplex Red, while alkaline phosphatase catalyzes ascorbic acid 2-phosphate, enabling a sensitive ratiometric fluorescence signal readout.

For CAT-like activity, the catalytic conversion of H_2_O_2_ into H_2_O and O_2_ makes these nanozymes particularly useful in disease therapy for mitigating oxidative stress and alleviating tissue hypoxia (Figure 1C) [49]. For instance, Feng et al. [50] synthesized an Ir@liposome nanozyme that exhibits effective near-infrared (NIR) responsive CAT-like activity towards H_2_O_2_. In in vivo experiments, the results demonstrated that the Ir@liposome nanozyme efficiently accumulated in tumors and substantially alleviated tumor hypoxia via H_2_O_2_ decomposition, proving its potential utility in cancer radiotherapy. SOD-like nanozymes are a class of nanomaterials capable of scavenging superoxide anions (•O_2_^−^) and converting them into H_2_O_2_ and O_2_ (Figure 1D) [51]. Similarly, since excessive •O_2_^−^ accumulation in vivo can induce tissue damage and associated inflammation, SOD-like nanozymes also hold significant potential for treating oxidative stress-related diseases due to their anti-inflammatory and antioxidant properties. Gao et al. [52] reported a carbon dot SOD-like nanozyme with catalytic activity exceeding 10,000 U/mg, which is comparable to that of natural SOD enzymes. This carbon dot SOD-like nanozyme effectively protected neuron cells in an ischemic stroke male mice model, demonstrating the therapeutic potential of carbon dots (CDs) in oxidation stress-related disorders.

### 2.2. Types of Nanozymes Used in Multiplex Signal Lateral Flow Immunoassays 

**Figure 2:**
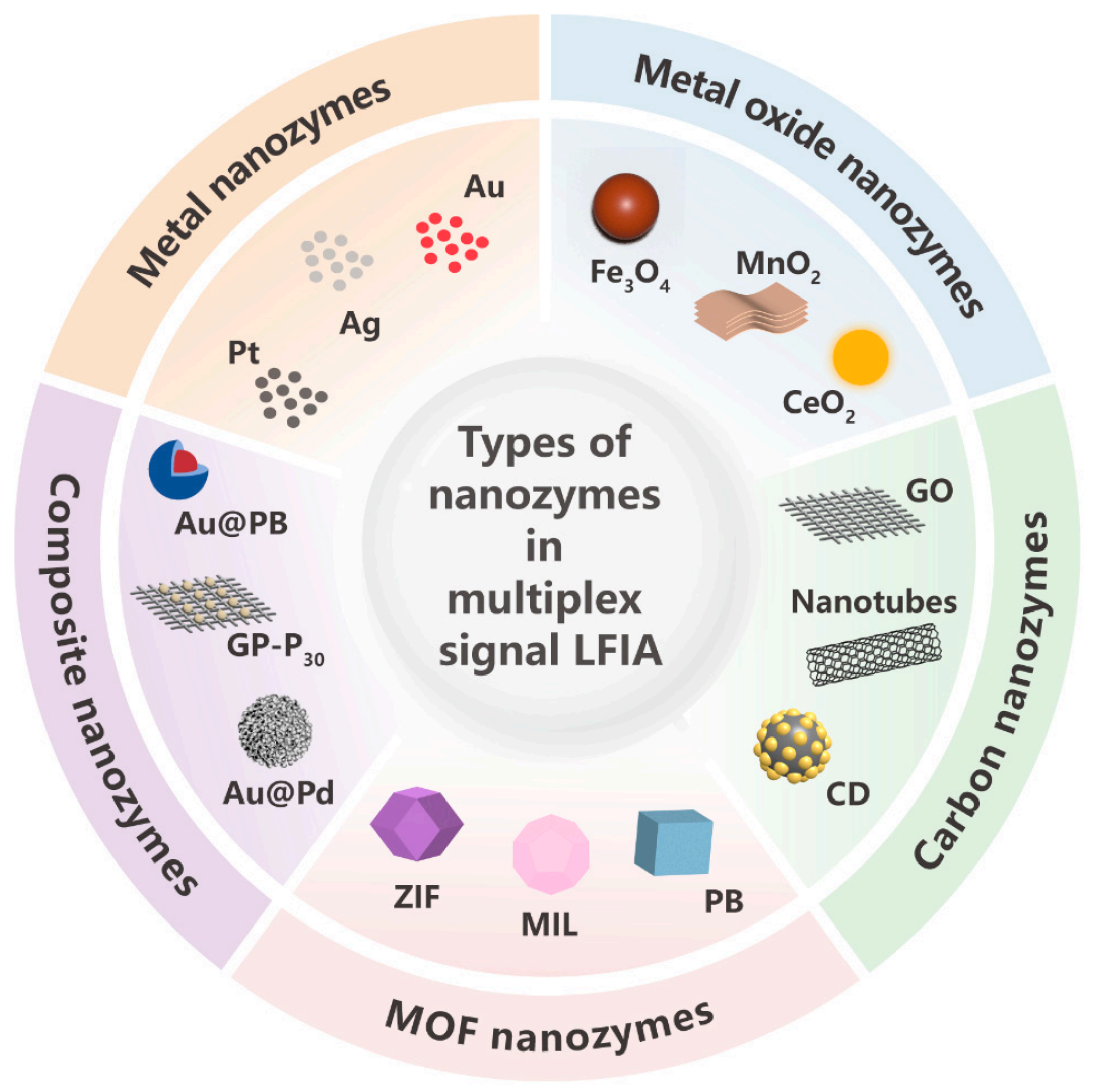
Types of nanozymes used in multiplex signal LFIA.

Owing to their unique enzyme-like catalytic activity, high stability, excellent structural tunability, and cost-effectiveness, nanozymes have emerged as promising alternatives to conventional GNP labels in LFIA. In addition to their catalytic functions, nanozymes may also possess multifunctional properties, such as superparamagnetic, visible-region light absorption, fluorescence, photothermal conversion, and SERS activity, making them particularly attractive for the development of dual- or multiple-mode LFIAs [53]. According to material composition, the nanozymes used in multiplex signal LFIAs can be categorized into metal nanozymes, metal oxide nanozymes, carbon nanozymes, MOF nanozymes, and composite nanozymes (Figure 2) [34,54]. The following sections provide a detailed discussion of each category and representative applications in multiplex signal LFIA.

#### 2.2.1. Metal and Metal Oxide Nanozymes

Metal nanozymes contain at least one metal element that serves as the catalytic active center [55]. Based on discrepancies of metal elements, they can be further divided into monometallic and metal alloys. Due to their excellent catalytic activity and stability, metal-based nanozymes are widely used in LFIAs. Liu et al. [56] designed an Fe-tannic acid (FTAN) nanozyme via facile chelation of Fe with TA. This FTAN exhibited superior POD-like activity, with a K_m_ of 0.152 mM and a V_max_ of 16.42 × 10^−8^ M for TMB. It enabled a colorimetric signal output through its native color while also catalyzing TMB to generate a catalytic amplified colorimetric signal, constructing a colorimetric/catalytic-enhanced colorimetric LFIA for RAC and clenbuterol (CLE) detection. Compared with transition metals such as Fe-, Co-, Mn-, and Cu-based nanozymes, noble metal (e.g., Au, Ag, Pt, Pd, Ru, Rh, and Ir)-based nanozymes exhibit superior catalytic efficiency due to their unique electronic configurations, which facilitate rapid electron transfer and lower activation energy barriers. Wang et al. [57] synthesized Au@Pt nanozymes with six Pt layers, which showed robust POD-like activity with a K_m_ of 0.41 ± 0.04 M, a catalytic constant (K_cat_) of 1.41 × 10^5^ s^−1^, and a catalytic efficiency (K_cat_/K_m_) of 3.40 × 10^5^ s^−1^ M^−1^, indicating higher substrate affinity and catalytic activity than HRP. This enabled its use as a novel signal label in LFIA for ofloxacin detection.

Metal oxide nanozymes are also commonly used as labels in LFIAs for chemical contaminants detection. Compared with metal nanozymes, metal oxide nanozymes are easier to synthesize and more cost-effective. As the first reported POD-like nanozyme, Wang et al. [58] synthesized an Fe_3_O_4_ nanozyme with immunomagnetic capture and enrichment capability as well as POD-like activity for aflatoxin B1 (AFB1) detection in LFIA. During detection, this nanozyme enabled target enrichment via magnetic separation and generated both an intrinsic colorimetric signal and a catalytic improved colorimetric signal through TMB oxidation. In addition to POD-like metal oxide nanozymes, OXD-like metal oxide nanozymes have also been reported for LFIA development. Cai et al. [59] utilized MnO_2_ nanosheets as a label in LFIAs, which possess strong OXD-like activity with K_m_ and V_max_ of 0.114 mM and 1.46 × 10^−7^ M/s toward TMB. Based on the MnO_2_-TMB catalytic amplification system, a highly sensitive and ultrawide linear range catalytic amplified colorimetric LFIA was also established for AFB1 *on-site* detection. Nevertheless, the extremely high surface free energy of metal and metal oxide nanozymes causes severe aggregation and poor dispersion stability, which limits their practical application in LFIAs.

#### 2.2.2. Carbon Nanozymes

Carbon nanozymes are a class of carbon-based nanomaterials with enzyme-like activity, which are composed of non-metal elements [60]. They mainly include graphene, graphene oxides (GOs), CD, graphene quantum dots, carbon nanotubes, and fullerenes. However, compared with other nanozymes, carbon nanozymes offer advantage of low cost, but their catalytic activity is relatively low. Therefore, they are often integrated with other materials to form composites for improved performance in LFIA. For example, Zheng et al. [61] constructed a GO/Au-AuPt nanozyme by assembling Au and AuPt nanoparticles on GO nanosheets. In this composite, GO provides a large surface area and stability, Au nanoparticles enhance colorimetric capability, and AuPt generates superior POD-like activity. This design successfully developed a colorimetric/catalytic-enhanced colorimetric dual-signal output LFIA capable of simultaneously detecting gentamicin (GM), CLE, and RAC residues. Similarly, Zhang et al. [62] also used GO nanosheets as a carrier to load Pt and AuIr nanoparticles for a GO-Pt_30_-AuIr nanozyme synthesis. This nanozyme exhibited K_m_ and V_max_ values of 0.240 mM and 3.602  ×  10^−8^ M/s, showing enhanced POD-like catalytic performance compared with GO-Pt_30_ nanozyme. Based on this GO-Pt_30_-AuIr nanozyme, a dual colorimetric/catalytic signal readout LFIA was established for heavy metal Cd^2+^ and pesticide acetamiprid (ACE).

#### 2.2.3. MOF Nanozymes

MOF nanozymes are porous crystalline materials formed by the coordination of metal ions with organic ligands [63]. Their highly porous structures, large specific surface areas, and tunable active sites make them widely applied in gas adsorption, energy storage, and analytical applications [64]. Hu et al. [65] synthesized a Prussian blue MOF nanozyme via a facile hydrothermal method as a dual-functional label. Based on its natural color for visual readout and POD-like activity to TMB for catalytic signal amplification, a colorimetric/catalytic amplified colorimetric LFIA was fabricated for the detection of norfloxacin in animal-derived foods. Owing to its inherent colorimetric properties, high POD-like activity, and excellent biocompatibility, Liu et al. [66] also used a Prussian blue MOF nanozyme to construct a dual-readout LFIA for the simultaneous detection of RAC and CLE. Through the colorimetric signal magnification achieved by catalyzing TMB oxidation, this dual colorimetric readout LFIA displayed significantly improved precision and a broadened detection range. However, MOF nanozymes still face challenges in precisely controlling morphology and particle size, as well as limited interference resistance in complex food matrices, which hinders their practical application in LFIAs.

#### 2.2.4. Composite Nanozymes

To meet the diverse demands of multiplex signal LFIA development, integrating different nanozymes into composites has become a practical strategy. This approach synergistically strengthens catalytic activity and enriches functional properties, overcoming the limitations of single nanozymes in sensitivity and detection modes [67]. Pt nanoparticle nanozymes have gained considerable attention due to their outstanding catalytic properties. However, they possess high surface energy, resulting in severe agglomeration in practical applications. Thus, to improve stability and catalytic activity, Du et al. [68] employed two-dimensional Ni(OH)_2_ nanosheet as a carrier to disperse Pt nanoparticles, designing a Pt-Ni(OH)_2_ composite nanozyme. The Ni(OH)_2_ nanosheet provides a large surface area with abundant catalytically active sites, while the strong interaction and synergistic effect between Ni(OH)_2_ and Pt nanoparticles favor substrate adsorption binding energy, achieving improved catalytic performance. Based on this Pt-Ni(OH)_2_ composite nanozyme, a smartphone-assisted colorimetric/catalytic-enhanced colorimetric LFIA was established for *on-site* and highly sensitive detection of acetochlor and fenpropathrin residues. In contrast, Sun et al. [69] used a bimetallic ZrFe-MOF as the loading carrier for Pt nanoparticles to prepare ZrFe-MOF@Pt nanocomposites. The ZrFe-MOF@Pt has excellent POD-like activity with a specific activity of 21.77 U/mg, which is approximately 103.79-fold higher than ZrFe-MOFs, indicating that Pt nanoparticle loading significantly boosted catalytic performance. Subsequently, a colorimetric/fluorescent/catalytic fancified colorimetric multimodal LFIA was developed by integrating ZrFe-MOF@Pt with a smartphone for aflatoxin M1 qualification. Currently, composite nanozymes are typically assembled from multiple nanozymes via multiple step synthesis, which complicates the preparation process and compromises structural stability. Moreover, effectively integrating different types of nanozymes to synergistically enhance overall catalytic activity remains a significant challenge.

## 3. Signal Combination Strategies in Nanozyme-Driven Multiplex Signal LFIA

Conventional LFIA relies on the accumulation of GNP immunoprobes on the T line for a single colorimetric signal readout, which suffers from poor sensitivity and compromised accuracy. In contrast, nanozyme-based multiplex signal LFIAs can simultaneously generate two or more independent and complementary signals (e.g., colorimetric, fluorescent, photothermal, or SERS) from a single T line. This multiple signal readout approach enables mutual signal enhancement and self-calibration, substantially improving LFIA sensitivity and reliability. The specific signal combinations in nanozyme-driven multiplex signal LFIA mainly include colorimetric/catalytic-enhanced colorimetric, colorimetric/chemiluminescent, colorimetric/fluorescent/catalytic-enhanced colorimetric, colorimetric/catalytic-enhanced colorimetric/photothermal, and colorimetric/catalytic-enhanced colorimetric/SERS [70,71,72,73]. The features of each signal combination in nanozyme-driven multiplex signal LFIAs are systematically summarized in Table 1.

### 3.1. Colorimetric/Catalytic-Enhanced Colorimetric Strategy

The colorimetric/catalytic-enhanced colorimetric strategy is the most straightforward approach in nanozyme-based multiplex signal LFIA. In this system, nanozyme-derived immunoprobes with intrinsic color offer an immediate qualitative readout by accumulation at the T line. Moreover, the catalytic activity of these immunoprobes enables catalysis of chromogenic substrates such as TMB, 3,3′-diaminobenzidine (DAB), or 3-amino-9-ethylcarbazole (AEC) to generate another more intense colorimetric signals after migration [74]. This additional catalysis-generated signal significantly amplifies the original accumulating colorimetric signal, resulting in improved sensitivity and broader linear range compared with conventional GNP-based LFIA. Thus, the colorimetric/catalytic-enhanced colorimetric mode offers a simple, cost-effective, and reliable approach to reinforce conventional LFIA performance.

### 3.2. Colorimetric/Fluorescent/Catalytic-Enhanced Colorimetric Strategy

Fluorescent signals exhibit superior sensitivity to colorimetric signals due to a higher signal to noise ratio. Therefore, the colorimetric/fluorescent/catalytic-enhanced colorimetric strategy has also been applied in nanozyme-based multiplex signal LFIA. In this LFIA system for chemical contaminants, fluorophores such as quantum dots or fluorescent microspheres are typically immobilized on the T line, and the accumulation of nanozyme labels generates a fluorescence signal through fluorescence quenching via the inner filter effect or fluorescence resonance energy transfer [69,71]. Similar to the signal combinations described above, the inherent color of nanozyme labels can be used for a visible color signal; their distinctive visible-region light absorption can quench the fluorescence of fluorophores for fluorescent signal readout, and their catalysis of a chromogenic substrate provides an additional colorimetric signal.

### 3.3. Colorimetric/Chemiluminescent Strategy

Additionally, besides catalyzing chromogenic substrates, nanozymes can also catalyze chemiluminescent substrates such as luminol-H_2_O_2_ to generate chemiluminescent signals. Owing to their self-luminescent feature, chemiluminescent signals eliminate interference from external light sources and offer higher sensitivity. In nanozyme-based colorimetric/chemiluminescent LFIA, the innate color of accumulated nanozyme labels provides a colorimetric signal, while their catalytic activity rapidly oxidizes chemiluminescent substrates on the T line, producing a sensitive chemiluminescent signal for accurate quantification [75]. In this signal combination, both the chemiluminescent and colorimetric signals can be easily recorded by a smartphone, facilitating *on-site* detection applications.

### 3.4. Colorimetric/Catalytic-Enhanced Colorimetric/Photothermal Strategy

In colorimetric/catalytic-enhanced colorimetric/photothermal multiplex readout LFIA, the nanozyme label simultaneously serves three functions: its native color generates an accumulation colorimetric signal, its catalytic activity catalyzes chromogenic substrate for an additional colorimetric signal, and its photothermal conversion capacity enables NIR absorption to produce a photothermal signal using a portable NIR laser [76,77]. The photothermal mode offers higher sensitivity due to its not being affected by color interference from sample matrices. Moreover, the three independent signals originating from the same label provide multiple cross-validations. The intrinsic colorimetric signal allows rapid visual inspection, the catalytic enforced signal improves sensitivity and extends linear range, and the photothermal mode enables precise quantification.

### 3.5. Colorimetric/Catalytic-Enhanced Colorimetric/SERS Strategy

The colorimetric/catalytic-enhanced colorimetric/SERS strategy exploits the SERS activity of nanozyme labels, combining it with their essential color and catalytic properties for multiplex signal readout in LFIA. Specifically, the inherent color of nanozyme labels mediates a colorimetric signal, and their catalysis of chromogenic substrates triggers a boosted colorimetric signal. For SERS signal, noble metal nanozymes with native localized surface plasmon activity, modified with Raman reporter molecules (e.g., dye molecules and thiolated aromatic molecules), generate SERS signals from the accumulated immunoprobes [78,79]. Compared with other optical signals, SERS signal eliminates background interference from biological matrices, offering extremely high sensitivity for precise quantification. This triple mode approach gathers the merits of amplified sensitivity and rapidity from each signal, significantly improving the performance of conventional LFIA.

## 4. Applications of Nanozyme-Based Multiplex Signal LFIA for Food Chemical Contaminants

The presence of chemical contaminant residues in food presents grave threats to consumer health and severely compromises the safety of the food supply chain. Thus, sensitive and accurate LFIA for *on-site* detection of such contaminants are critically needed to safeguard food safety and public health. Currently, nanozyme-based multiplex signal LFIA has been widely applied in the detection of chemical contaminants in food, including veterinary drugs, mycotoxins, pesticides, and other contaminants [56,62,68,80,81,82]. Table 2 summarizes the studies of nanozyme-based multiplex signal LFIA for detecting food chemical contaminants in various food matrices.

### 4.1. Veterinary Drugs

**Figure 3:**
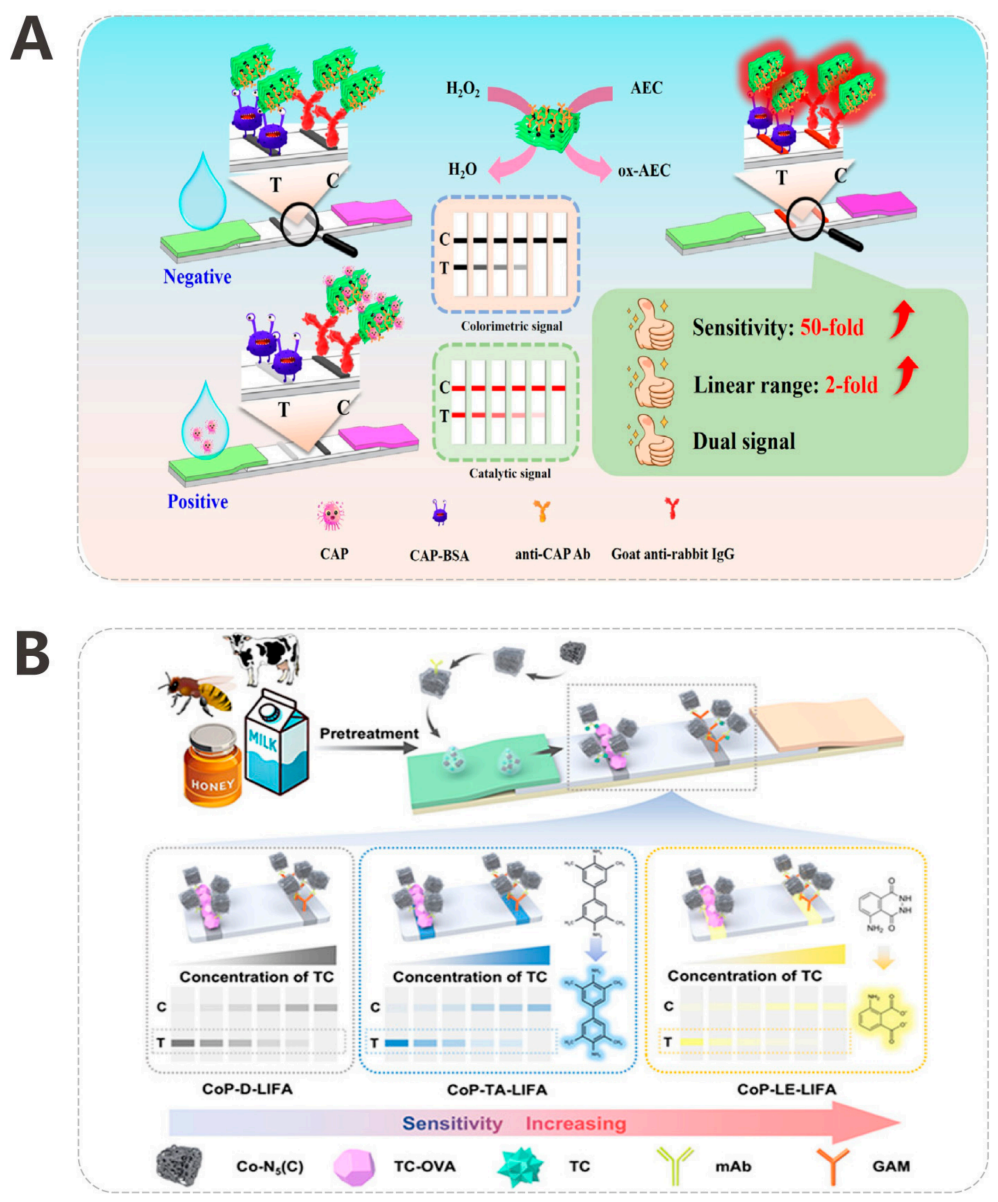
(**A**) Schematic of Ti_3_C_2_Tx@Pt composite nanozyme-driven dual-colorimetric LFIA for CAP detection. Open access article [88]. (**B**) Schematic of CoSAN-based colorimetric/catalytic-enhanced colorimetric/chemiluminescent triple-mode LFIA for TC detection. Reprinted with permission from ref. [94].

Veterinary drugs are a class of substances used for clinical diagnosis, prevention, and treatment of animal disease, as well as for improving animal production performance [119]. They mainly include antibiotics, antiparasitics, and growth promoters. When these substances are illegally used or abused, their residues accumulate in animal-derived foods, posing direct risks to consumers. Currently, nanozyme-based multiplex signal LFIA provides robust support for the screening of veterinary drug residues. Hu et al. [89] designed ultra-small Cu-Au bimetallic nanozymes (USCGs) with innate POD-like activity, which served as the label in a colorimetric/catalytic amplified colorimetric dual-signal LFIA for CLE detection. In this LFIA, the USCGs catalyze TMB-H_2_O_2_ to produce a catalytic amplified color signal, which combines with the pre-catalysis accumulated colorimetric signal to enable dual mode determination. Under optimal conditions, the LFIA achieved a limit of detection (LOD) in catalytic-amplified mode of 0.03 ng/mL, 6-fold and 2-fold lower than conventional GNP-based LFIA and pre-catalysis colorimetric mode, respectively. The assay time was only 7 min with a catalytic step of 80 s, offering an advanced sensing platform for CLE detection in food samples. Theoretically, multiple noble metals-based composite nanozymes exhibit higher catalytic activity than those synthesized using a single noble metal with a transition metal. Hence, Bai et al. [84] synthesized a trimetallic dPdPt-Ir nanozyme featuring abundant catalytic sites. Taking advantage of its enforced POD-like activity, the dPdPt-Ir nanozyme catalyzed the AEC-H_2_O_2_ substrate system to produce a strongly brown colorimetric signal. In the pre-catalysis colorimetric mode based on dPdPt-Ir aggregation, the LOD for CLE was 39.28 pg/mL. After post-catalysis magnification via AEC oxidation, the LOD reached 7.15 pg/mL with a linear range of 0.01–100 ng/mL, representing a 5.5-fold and 47.7-fold sensitivity improvement compared to pre-catalysis colorimetric readout and conventional GNP-based LFIA (LOD: 341.32 pg/mL), respectively. Moreover, the total assay time was only 20 min, recoveries in real samples ranged from 80.37 to 111.69%, and relative standard deviations were below 6.91%, verifying the practicality of this dual-signal LFIA. In addition to the detection of β-agonist residues, Lin et al. [88] constructed a Ti_3_C_2_Tx@Pt composite nanozyme as a label for dual-colorimetric LFIA for CAP detection. The Ti_3_C_2_Tx nanosheets provide a large specific surface area for Pt NP loading, exhibiting heightened POD-like activity. After capture on the T line, the nanozyme-derived immunoprobes not only provide an initial black color signal but also catalyze AEC to generate an extra colorimetric signal for sensitivity boosting. After optimization, the initial colorimetric mode exhibited an LOD of 0.01 μg/kg in milk, chicken, and fish with a linear range of 0.0125–0.5 μg/kg. For the ACE magnified colorimetric mode, the LOD was also 0.01 μg/kg, but the linear range broadened to 0.0125–1.0 μg/kg. Compared with the reported GNP-based LFIA, this dual-colorimetric LFIA achieved a 50-fold sensitivity boost, attributed to the large specific surface area of Ti_3_C_2_Tx@Pt, which allows more antibodies to be loaded for detection (Figure 3A).

As mentioned above, chemiluminescent signals generally offer higher sensitivity than colorimetric signals. Thus, multiplex LFIA with chemiluminescent signal readout is highly attractive. Huang et al. [94] synthesized a cobalt single-atom nanozyme (CoSAN) via a dual-template pyrolysis strategy. This CoSAN exhibits outstanding catalytic activity, catalyzing both the TMB-H_2_O_2_ and luminol-H_2_O_2_ systems for dual catalytic colorimetric and chemiluminescent signal outputs. Using its gray color and catalytic magnification, a colorimetric/catalytic intensified colorimetric/chemiluminescent triple-mode LFIA was developed for TC detection. The LODs of this LFIA in colorimetric, catalytic-intensified colorimetric, and chemiluminescent modes were 0.091 ng/mL, 0.062 ng/mL, and 0.056 ng/mL in milk and honey samples, achieving 43-, 63-, and 70-fold sensitivity improvements compared to GNP-based LFIA (Figure 3B). Moreover, to enable more signals for cross-validation in chemical contaminant LFIA, Luo et al. [93] constructed a ruthenium–polydopamine (Ru-PDA) hybrid nanozyme that integrates four functionalities: high molar extinction coefficient, broad visible-region absorption, enforced POD-like activity, and strong photothermal conversion capability. Based on this multifunctional nanozyme, a colorimetric/fluorescent/catalytic-enhanced colorimetric/photothermal multiplex LFIA was developed for xylazine detection. The corresponding LODs were 0.032 ng/mL, 0.0041 ng/mL, 0.014 ng/mL, and 0.0087 ng/mL, representing up to 11.7-fold improvements in sensitivity over conventional GNP-based LFIA, along with wider linear ranges. This work highlights the practicality of nanohybridization strategy in designing versatile signal labels and advancing multimodal LFIA applications.

### 4.2. Mycotoxins

**Figure 4:**
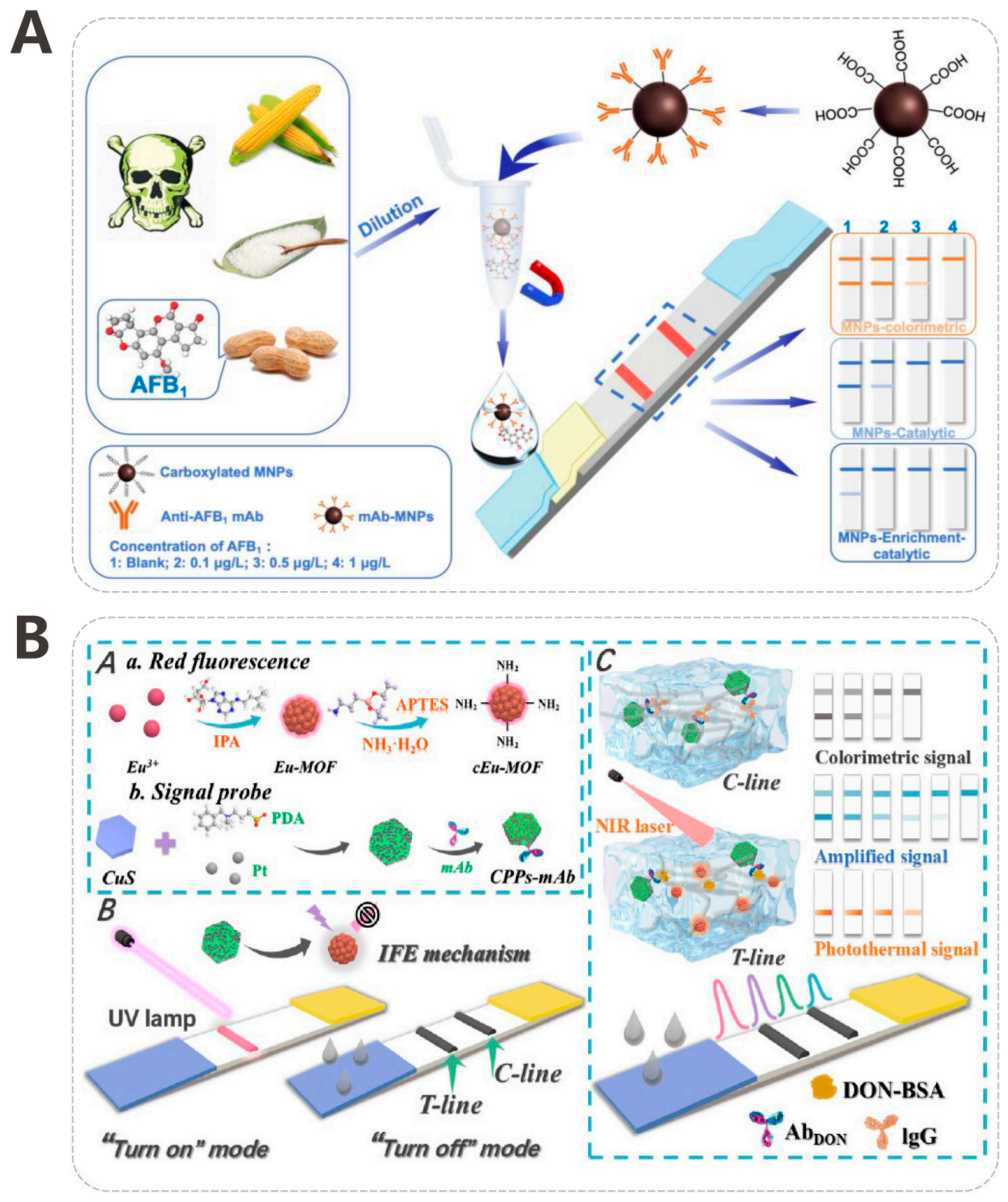
(**A**) MNPs-based colorimetric/catalytic/magnetic enrichment plus catalysis triple-mode LFIA for detection of AFB1. Open access article [58]. (**B**) CPP nanozyme-based colorimetric/fluorescence quenching/catalytic-enhanced colorimetric/photothermal multiplex LFIA for DON detection. (*A*) Synthesis scheme; Development of bimodal (*B*) and multisignal (*C*) immunochromatographic sensors. Reprinted with permission from ref. [104].

Mycotoxins are small-molecule secondary toxic metabolites produced by fungi such as Aspergillus, Fusarium, Alternaria, Verticillium, and Penicillium. They can contaminate throughout each stage of the food chain and pose serious health threats to humans and livestock [120]. Therefore, the development of rapid and reliable multiplex signal LFIAs for mycotoxins is urgently needed for food safety control. Xuan et al. [98] synthesized a novel CuS@Au-Pt (CAP) biomimetic nanozyme with three synergistic advantages: increased colorimetric brightness, excellent POD-like activity, and improved electron-transport efficiency. This CAP nanozyme used as a label in an LFIA provides a dual readout: a direct accumulation color signal followed by a TMB-catalyzed amplified color signal. For BA detection, this dual-mode LFIA achieved LODs of 0.66 ng/mL (colorimetric mode) and 1.05 ng/mL (catalytic-enhanced colorimetric mode), which are 3.25 times and 2.05 times improvements compared to GNPs-LFIA (2.15 ng/mL). The CAP-based dual-signal LFIA was successfully applied to detect BA in tremella, corn flour, and millet flour samples with satisfactory recovery. Meanwhile, Wang et al. [58] designed spherical carboxylated magnetite magnetic nanoparticles (MNPs) via co-precipitation method. The resulting MNPs not only enable the enrichment of analytes through inherent magnetic properties but also exhibit excellent POD-like activity to catalyze TMB-H_2_O_2_ for further signal amplification. Based on these MNPs, a triple colorimetric mode LFIA for AFB1 was established, which integrates colorimetric, catalytic, and magnetic enrichment plus catalytic amplified colorimetric signal readout. The LODs in three modes are 0.34 μg/L, 0.17 μg/L, and 0.023 μg/L, respectively. Notably, compared to the initial colorimetric mode, sensitivity increased 2-fold after catalysis and further improved by 14.8-fold after magnetic enrichment plus catalysis. Additionally, recovery rates in rice, corn, and peanuts ranged from 70 to 134.43%, verifying the feasibility of the MNPs-based triple-signal LFIA (Figure 4A). Both of the above dual-mode LFIAs can only be used for a single mycotoxin detection. Given the frequent co-contamination of multiple mycotoxins, there is a need to develop an LFIA capable of simultaneous quantification of multiple mycotoxins. Zhou et al. [99] innovatively designed a POD-like microbial nanozyme (ESi-AuPt) by coating *Escherichia coli* with a SiO_2_ interlayer and modifying it with catalytic AuPt nanoparticles, serving as a label for a dual-mode LFIA for AFB1, ZEN, and FB1 detection. In this LFIA, ESi-AuPt nanozyme exhibits superior inherent color and boosted catalytic activity to AEC-H_2_O_2_ substrate, producing colorimetric and catalytic colorimetric signals for dual readout. After optimization, the LODs in colorimetric mode for FB1, AFB1, and ZEN were 0.0077 ng/mL, 0.0032 ng/mL, and 0.016 ng/mL. In catalytic colorimetric mode, the LODs for the three mycotoxins achieved 0.0061 ng/mL, 0.0016 ng/mL, and 0.0054 ng/mL, with linear ranges improved by 9–250-fold compared to colorimetric mode. The recoveries for the three mycotoxins in rice, corn, and lake water samples were satisfied in 85.9–116.1%.

The noble metal nanozyme not only commonly possesses high catalytic activity but also exhibits distinct localized surface plasmon activity, utilized for photothermal and SERS signal output [121]. Xie et al. [102] used Au-Pt bimetallic nanoflowers (AuPt NFs) as the label in a triple-mode LFIA for AFB1 analysis. Compared with GNPs, AuPt NFs exhibit superior optical properties, excellent POD-like activity, and strong photothermal conversion ability, allowing colorimetric, catalytic-enhanced colorimetric, and photothermal signal readouts. Both colorimetric and photothermal modes showed linear ranges of 0.05–1 ng/mL with LODs of 0.05 and 0.04 ng/mL, respectively. The catalytic caused the colorimetric mode to achieve a wider linear range (0.01–1 ng/mL) and a lower LOD of 0.01 ng/mL. This study demonstrates the application of AuPt NFs in novel photothermal signal-based multiplex LFIA. Owing to the large specific surface area, two-dimensional layered nanozymes expose greater surface catalytic sites and enable higher antibody loading, achieving superior detection performance. Xuan et al. [104] designed an “all-in-one” two-dimensional CuS@PDA (CPP) nanosheet with broad-spectrum absorption, POD-like activity, and exceptional photothermal conversion capacity. Based on the above multifunction, the CPP nanozyme as an LFIA label can achieve colorimetric, fluorescence quenching, catalytic-enhanced colorimetric, and photothermal signal output. In this quadruple-signal LFIA, the LODs for DON in colorimetric, fluorescent, catalytic-boosted colorimetric, and photothermal modes were 0.032 ng/mL, 0.5 ng/mL, 0.021 ng/mL, and 0.056 ng/mL, respectively. Compared with GNPs-based LFIA, the sensitivity of colorimetric, catalytic-boosted colorimetric, and photothermal modes obtained 3.1, 4.7, and 1.75 times improvement, and the linear range improved over 1-fold. Recoveries of this multimodal LFIA in millet and maize samples ranged from 82.5 to 119.4%, exhibiting acceptable reliability and feasibility for DON detection in real samples. This multimodal platform integrates fluorescent signals for low-background quantification, colorimetric detection for rapid screening, and photothermal readout for high sensitivity, significantly overcoming the poor sensitivity and external interferences of conventional single-signal LFIA (Figure 4B).

### 4.3. Pesticides

The widespread use of pesticides in agriculture has severely increased risks to human health and ecological security. Notably, even trace pesticide residues in agricultural products can accumulate in the human body through ecosystem circulation and cause serious poisoning events [122]. Therefore, establishing a reliable and sensitive multimodal LFIA for pesticide residue detection in food samples is also urgently needed. Zhang et al. [107] synthesized PtPdRu NFs with natural colorimetric capability and exceptionally high POD-like activity (23.7-fold higher than Pt NFs). Employing this PtPdRu NF as a bifunctional label, a portable triple-colorimetric sensing platform for thiamethoxam was constructed by integrating LFIA with a smartphone. This multimodal LFIA platform enables three readout modes: visual color readout with vLOD of 5 ng/mL, quantitative based on gray value with LOD of 0.13 ng/mL, and TMB catalysis for quantitation with LOD of 0.03 ng/mL. The catalytic-intensified colorimetric mode provides a 4.3-fold sensitivity improvement over the pre-catalysis mode. Excellent agreement with instrumental methods and satisfactory recoveries in cowpea samples confirmed the practicality of this LFIA. For pesticide detection, a conventional LFIA usually requires complex sample pretreatment, which significantly slows down *on-site* screening efficiency. Zhai et al. [106] synthesized a core-shell Fe_3_O_4_-MOF-Pt composite nanozyme by successively modifying Fe_3_O_4_ nanoparticles with MIL-100(Fe) MOF and chloroplatinic acid. In an LFIA for CAR detection, this Fe_3_O_4_-MOF-Pt label possesses magnetic separation for target enrichment, POD-mimic activity to AEC-H_2_O_2_ system for signal expansion, and essential color for direct visual readout. The vLODs for CAR in native color mode and AEC catalysis-advanced colorimetric mode were 0.5 ng/mL and 0.15 ng/mL. The magnetic property simplifies sample pretreatment, resulting in improved detection efficiency. The spiked vegetable samples yielded recoveries of 91.40–102.40%, confirming that the dual-signal LFIA can accurately and reliably detect CAR residues in real samples.

**Figure 5:**
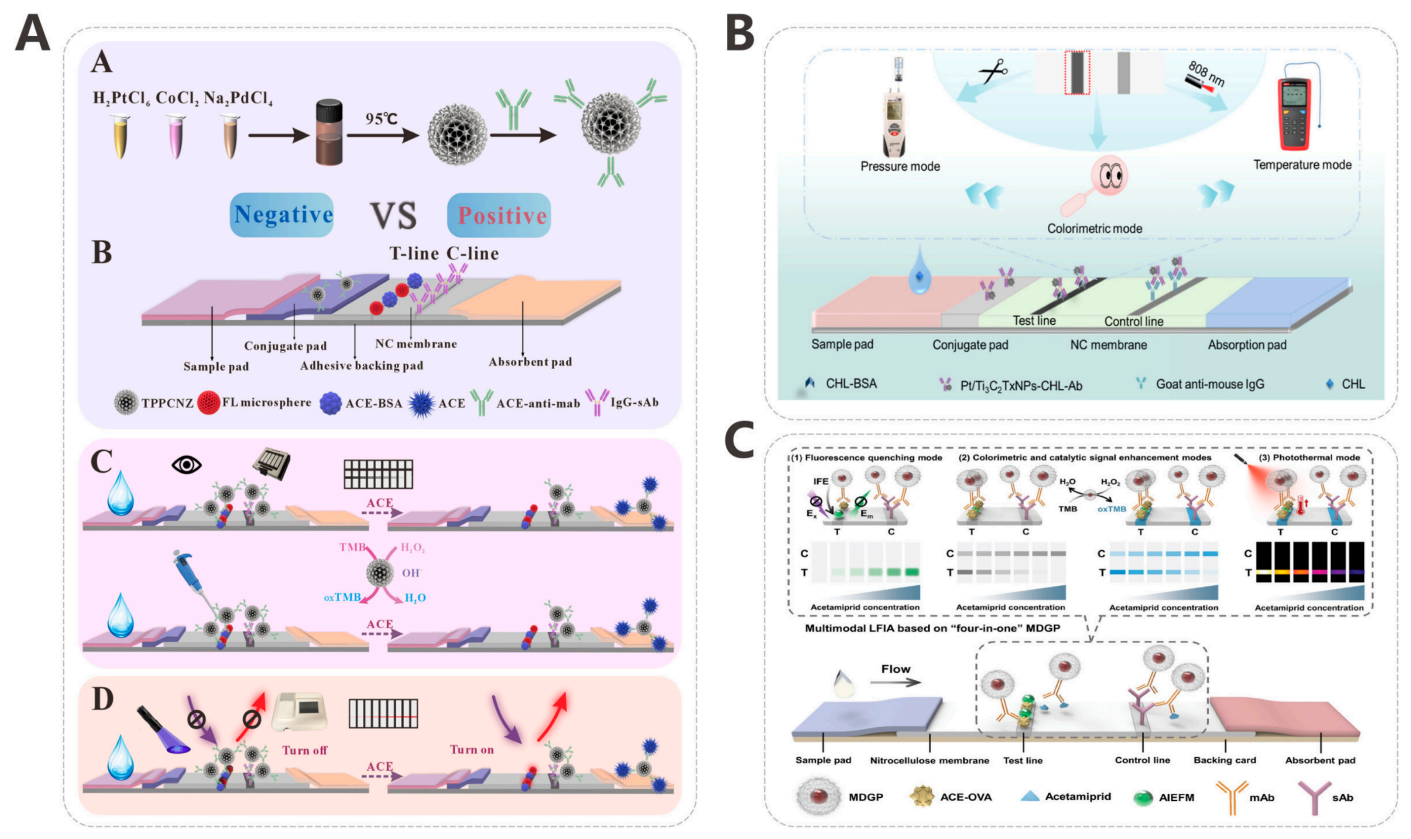
(**A**) A PtPdCo nanozyme-facilitated colorimetric/catalytic-enhanced colorimetric/fluorescent multimodal LFIA for highly sensitive ACE detection. (*A*) Preparation of immunoprobes; (*B*) Assembly of LFIA; (*C,D*) Schematic illustration of the detection principle. Reprinted with permission from ref. [109]. (**B**) A Pt/Ti_3_C_2_Tx composite nanozyme-based colorimetric/pressure/photothermal triple-mode LFIA for CHL detection. Reprinted with permission from ref. [111]. (**C**) An Au@Pt nanozyme-based colorimetric/catalytic-enhanced colorimetric/fluorescent/photothermal quadruple-signal LFIA for ACE detection. Reprinted with permission from ref. [108].

In addition to dual-colorimetric signal readout, LFIA integrated with more output signal types has also been developed for pesticide residue detection. Li et al. [109] fabricated a trimetallic PtPdCo mesoporous nanozyme label in a multimodal LFIA to detect ACE. This PtPdCo nanozyme exhibits three distinct signal transduction capabilities: strong colorimetric response, POD-like catalysis, and fluorescence quenching. Based on these multifunctional properties, this multimodal LFIA provides colorimetric (LOD of 1.7 pg/mL), catalytic-amplified colorimetric (LOD of 4.9 pg/mL) and fluorescent (LOD of 0.0115 ng/mL) readouts, outperforming reported GNP-based LFIA for ACE (LOD of 10 ng/mL). In real food samples, this triple-mode LFIA showed satisfactory recoveries of 72.47–120.86%, demonstrating its practical potential for rapid pesticide residue screening (Figure 5A). In addition to transduced signals such as light, electricity, and heat, new types of signals, such as pressure signal, have also been employed for multimodal LFIA construction. For instance, Xue et al. [111] synthesized a Pt/Ti_3_C_2_Tx composite nanozyme by loading Pt nanoparticles onto single-layer Ti_3_C_2_Tx nanosheets. This design endows the composite with deep color for visual readout, excellent CAT-like activity to catalyze H_2_O_2_ decomposition for pressure signal output, and high photothermal conversion efficiency for temperature signal output. Based on these distinct functions, a triple-mode LFIA was developed for CHL detection, offering LODs in colorimetric, pressure, and photothermal modes of 5 ng/mL, 0.04 ng/mL, and 0.09 ng/mL, respectively. The pressure and photothermal modes enable precise quantitation by portable devices, while the colorimetric mode facilitates rapid visual screening, enhancing accuracy and applicability. Recoveries in spiked herbal samples ranged from 84.0 to 114%, fulfilling practical analysis requirements (Figure 5B). To merge colorimetric, catalytic-amplified colorimetric, photothermal, and fluorescent signals in a single LFIA, Wang et al. [108] constructed a dandelion-like Au@Pt nanozyme featuring a broad absorption spectrum, high POD-like activity, dual spectral-overlapped fluorescence quenching capability, and outstanding photothermal conversion ability. Combining this with an aggregation-induced emission fluorescent microspheres as the fluorophore, a quadruple-signal LFIA that simultaneously provides optical color signal, catalytic-improved color signal, fluorescent signal, and a photothermal signal readout was established for ACE detection. In optimized conditions, this multimodal LFIA achieves LODs ranging from 0.008 ng/mL to 0.098 ng/mL across four modes, showing a 56.2-fold sensitivity improvement in photothermal mode compared to GNP-based LFIA for ACE. The spiked test in apple samples presented in 85.4–109.4%, verifying the feasibility of this LFIA in actual detection scenarios (Figure 5C).

### 4.4. Other Contaminants

**Figure 6:**
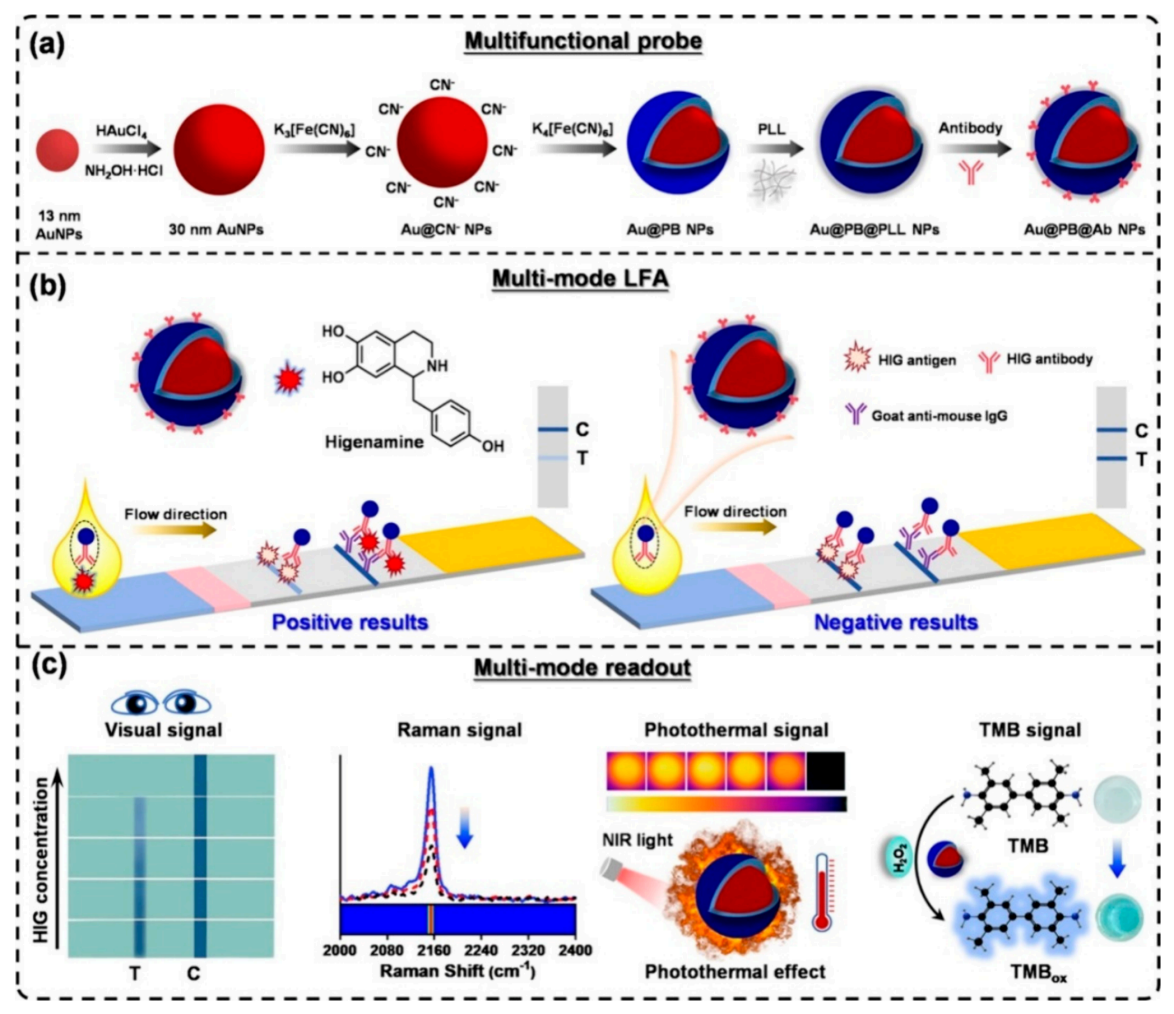
An Au@PB nanoparticle-based colorimetric/catalytic-enhanced colorimetric/photothermal/SERS quadruple-signal LFIA for HIG detection. (**a**) Preparation Process; (**b**) Principle of LFIA detection; (**c**) Visual, Raman, photothermal, and colorimetric readout signals. Reprinted with permission from ref. [118].

Food additives are substances added to foods to improve appearance, fragrance, taste, or shelf life and are widely applied in the food industry [123]. However, abuse of permitted additives and use of illegal additives can lead to food safety incidents and threaten the health of food consumers. Thus, the development of rapid and accurate multiplex LFIAs for relevant food additives is of critical importance to food safety monitoring. Pan et al. [112] synthesized an Au@Pt POD-like nanozyme as a signal label for a dual colorimetric LFIA to detect antipyrine in herbal tea and surface water. After binding on the T line, the Au@Pt immunoprobes produce a visible color signal based on their intrinsic black color, and a catalytic-amplified colorimetric signal was triggered by catalysis of TMB-H_2_O_2_. This catalytic amplification lowered the LOD of conventional GNPs-based LFIA from 4.59 ng/mL (herbal tea) and 7.22 ng/mL (surface water) to 3.03 ng/mL and 2.0 ng/mL, satisfying the requirement of antipyrine detection in tea samples. Spiked results showed recoveries of 87.9 –111.1 % in herbal tea and surface water samples, proving its accuracy in real sample analysis. Building on the colorimetric/catalytic-enhanced colorimetric mode, additional signal channels have been introduced to develop a multimodal LFIA system. Regarding the illegal use of sedatives in aquatic products, Zhang et al. [117] designed an AuPt-doped copper hexacyanoferrate nanozyme (AuPt@Cu-HCF) as a multifunctional signal reporter. Its intrinsic color provides visual readout, NIR absorption enables photothermal signal, catalytic activity drives TMB oxidation for catalytic colorimetric amplification, and the combination of TMB catalysis with NIR irradiation produces a catalytic-intensified photothermal effect. Based on these, a “four-in-one” cascade LFIA was established for diazepam detection in lake water and fish, achieving LODs of 0.82 ng/mL (colorimetric mode), 12.82 pg/mL (photothermal mode), 12.26 pg/mL (catalytic colorimetric mode), and 4.43 pg/mL (catalytic photothermal mode). This multimodal LFIA could complete detection within 20 min and show acceptable recoveries in lake water and fish samples, offering a tunable platform suitable for various detection scenarios. Ding et al. [118] designed an Au@PB nanoparticle with a core-shell structure, in which the Au nanoparticle confers significant colorimetric and SERS responses, whilst the PB shell provides excellent POD-like activity and NIR absorption properties. Based on the Au@PB nanoparticle, a colorimetric/catalytic-enhanced colorimetric/photothermal/SERS quadruple-mode LFIA was established for HIG detection. The LODs in the four distinct modes were 1.07, 0.68, 0.71, and 0.01 ng/mL, respectively. Compared with the colorimetric mode, the additional signals enhanced sensitivity to varying degrees, and accuracy was improved through cross-validation of multiple signals, thereby delivering more robust analytical performance (Figure 6).

Heavy metal contamination originating from natural sources, industrial effluents, and agricultural activities presents a serious threat to food and environmental safety [124]. Consequently, to achieve rapid and accurate detection of heavy metal ions in food samples, establishing a novel LFIA is extremely needed. Bai et al. [87] fabricated an Au/Ir bimetallic modified SiO_2_ (Si@Au/Ir) nanozyme label that provides both a colorimetric signal and a catalytic magnification colorimetric signal via TMB-H_2_O_2_ oxidation in LFIA for Cd^2+^ detection. After optimization, the LODs of this dual colorimetric LFIA in colorimetric and catalytic-amplified colorimetric mode were 97.6 pg/mL and 0.65 pg/mL, with a broadly wide dynamic detection range of 0.0015–100 ng/mL. In detection of Cd^2+^ in milk, oats, soil, and river water, recoveries range from 90.78 to 105.25%, with relative standard deviation lower than 8.7%. Similarly, for Cd^2+^ detection in food and environmental samples, Zhang et al. [62] fabricated a GO-Pt_30_-AuIr nanozyme for direct colorimetric and catalytic-amplified colorimetric dual signal outputs in LFIA via its essential color and POD-like activity for TMB catalysis. For Cd^2+^ quantification, this dual-signal LFIA presented the LODs of 71.25 pg/mL and 7.02 pg/mL in colorimetric and catalytic-amplified mode, with 7.12-fold sensitivity enhancement compared to conventional LFIA. The practical application performance of this LFIA in dual mode was verified by corn, lettuce, lake water, and river water samples, with recoveries ranging from 81.2  to 108.9 %, providing a reliable tool for rapid screening of Cd^2+^ in various samples.

## 5. Conclusions and Future Perspectives

Chemical contaminants in food samples represent a large-scale and intensifying threat to public health within the global food supply. Consequently, establishing sensitive, accurate, and cost-effective on-site analytical methods is vital to safeguard consumer food safety. Currently, LFIA is one of the most commonly used techniques for the on-site monitoring of chemical contaminants in food. However, conventional GNPs-based single-signal LFIA suffers from inherent limitations such as poor sensitivity, inadequate stability, and accuracy. The synergistic combination of nanozymes with multiplex signal readout strategies has achieved substantial improvements in sensitivity, stability, accuracy, and precise quantitation for food chemical contaminants. This review systematically covers recent advances in nanozyme-based multiplex signal LFIA for the detection of chemical contaminants in food. The unique catalytic properties and classifications of nanozymes are first summarized. Based on the multifunctional properties of nanozymes, the emerging signal combination strategies in nanozyme-based multiplex signal LFIA have been detailed. In particular, the specific applications of nanozyme-based multiplex signal LFIA for food chemical contaminants detection are highlighted.

Despite the high attractiveness and rapidly growing research interest in nanozyme-based multiplex signal LFIA for food chemical contaminants detection, this field still faces several critical challenges that hinder its practical application.

(1) In nanozyme-based multiplex signal LFIA, current research has primarily focused on the POD-like nanozymes. In contrast, OXD-like nanozymes can directly catalyze substrate oxidation without the need to introduce unstable H_2_O_2_, simplifying the detection process and enhancing the convenience of *on-site* operations. However, research on this class of nanozymes is still in its infancy, and the variety of available options remains relatively limited. In addition, whether they are POD-like or OXD-like nanozymes, their overall catalytic activity is still generally inferior to that of natural enzymes, which limits further performance improvements in the LFIA system. Therefore, the rational design of nanozymes with high catalytic efficiency is key to enabling more favorable catalytic-enhanced multiplex signal LFIAs. In this regard, single-atom nanozymes represent a suitable and highly promising candidate, as their atomically dispersed active sites maximize atom utilization and allow finely tunable coordination environments to boost catalytic activity [125]. Furthermore, computer-based theoretical design strategies, such as machine learning, can provide atomic-level structure–function relationship analysis, guiding and accelerating the discovery of high catalytic activity nanozymes [36].
(2) The simultaneous output of diverse data from multiple channels in nanozyme-based multiplex signal LFIA complicates quantitative interpretation and impairs detection efficiency. Fortunately, the emergence and advancement of machine learning techniques provide an important tool for interpreting these output signals. Trained on large amounts of existing detection data, machine learning can accurately extract critical quantitative characteristic information while eliminating background interference from the T line. Furthermore, machine learning can accelerate the self-calibrating quantification process by fusing data from multiple signal channels, significantly improving the accuracy of LFIA based on different signal pairs. Several studies have already verified the practicality of machine learning in colorimetric/SERS and colorimetric/fluorescent multiplex signal LFIA for food chemical contaminants, achieving notable improvements in sensitivity, accuracy and detection efficiency [126,127,128].
(3) The integration of multiplex signals in LFIA system imposes higher requirements on readout devices, particularly in terms of miniaturization and portability, which are crucial for *on-site* detection. Although such multiplex LFIA systems have not yet been reported, multimodal immunoassays based on commercial portable devices such as glucose meters represent a promising solution [129,130]. Furthermore, smartphones with powerful processing units are emerging as interfaces for quantitative analysis. By being equipped with various miniaturized modular sensors (e.g., optical, thermal, and potentiostat sensors), they enable multiple detection modalities to be integrated into a single readout device, constituting another potential solution for multiplex signal LFIAs [131,132,133].
(4) The practical application of multiplex signal LFIA in complex food matrices is primarily hindered by matrix interference and the catalytic instability of nanozymes. Components such as fats, proteins, and polysaccharides may cause nonspecific adsorption or interfere with signal generation, compromising sensitivity and accuracy. Therefore, developing novel sample pretreatment techniques, such as miniaturized solid-phase extraction, is crucial for mitigating matrix interference [134]. Additionally, variations in pH and ionic strength across different detection environments can affect the catalytic stability of nanozymes, leading to inaccurate quantification. Consequently, strategies such as surface modification and structural encapsulation are feasible approaches to enhance the catalytic stability of nanozymes as signal labels in LFIAs within complex systems [135,136].

## Figures and Tables

**Table 1:** The signal combinations in multiplex signal LFIAs.

Multiplex Signal Types	Typical Nanozymes	Advantages	Disadvantages
Colorimetric/catalytic-enhanced colorimetric	Au@Ag PB AuPt	Visual signal amplification Cost-effective User-friendly operation	Color interferences Limited sensitivity Poor quantitative capability
Colorimetric/fluorescent/catalytic-enhanced colorimetric	Au@Pt ZrFe-MOF@Pt CPP	Suitable for complex samples Visual and sensitive quantification Wider dynamic range	Requires additional spectra devices Background fluorescence interferences Photobleaching phenomenon
Colorimetric/chemiluminescent	MnO_2_	No external excitation light required Low background interferences High sensitivity Wider dynamic range	Limited light-emission duration Poor stability Time-sensitive detection
Colorimetric/catalytic-enhanced colorimetric/photothermal	Fe_3_O_4_@PDA@Pt AuPt CoFe PBAs/WS_2_	High sensitivity Low background interferences Real-time monitoring	Requires additional NIR laser and thermal imager Affected by ambient temperature
Colorimetric/catalytic-enhanced colorimetric/SERS	Au@PB	Ultra-high sensitivity Strong anti-interference capability Fingerprinting capability	Requires extra SERS device Higher cost Poor reproducibility

**Table 2:** Applications of nanozyme-driven multiplex signal LFIA in food chemical contaminant detection ^a^.

Application	Nanozymes	Signals	Analytes	Samples	LODs	Linear Ranges	References
Veterinary drugs	Au@Ag NPs	Colorimetric/catalytic-enhanced colorimetric	Gatifloxacin	Chicken, turkey, rabbit, pork, beef, lamb	1.4 pg mL^−1^/0.8 pg mL^−1^	2–40 ng mL^−1^/- ^b^	[83]
	Au@Pt NPs		Ofloxacin	Chicken, fish	0.017 ng mL^−1^/0.018 ng mL^−1^	0.06–4.512 ng mL^−1^/0.079–12.387 ng mL^−1^	[57]
	dPdPt-Ir		CLE	Lake water, vegetable market wastewater, pork, milk	39.28 ng mL^−1^/7.15 pg mL^−1^	-/0.01–100 ng mL^−1^	[84]
	Fe-Au@Pt		GM, streptomycin, CLE	Honey, milk, pork	18.1, 16.1, 2.9 pg mL^−1^/10.1, 6.3, 1.1 pg mL^−1^	0.04–0.4, 0.013–0.13, 0.004–0.13 ng mL^−1^/0.04–11.1, 0.013–3.7, 0.004–11.1 ng mL^−1^	[85]
	FTAN		RAC, CLE	Pork, pork liver	-/0.015, 0.156 ng mL^−1^	0–0.6, 0–6 ng mL^−1^/0.2–1, 1–10 ng mL^−1^	[56]
	GO/Au-AuPt		GM, CLE, RAC	Pork, chicken, lake water, river water	11.24, 17.65, 16.86 ng mL^−1^/2.61, 3.61, 4.9 pg mL^−1^	-	[61]
	MPBN		RAC, CLE	Pork, mutton	1, 1 ng mL^−1^/0.12, 0.20 ng mL^−1^	0.5–3.5, 0.5–6 ng mL^−1^/1–6, 1–12 ng mL^−1^	[66]
	PB NPs		Furazolidone	Skimmed milk, chicken, pork	0.4 ng mL^−1^/0.33 ng mL^−1^	0.2–5 ng mL^−1^/0.2–10 ng mL^−1^	[86]
	PB NPs		Norfloxacin	Pork, chicken, beef, sea bass, shrimp	0.5 ng mL^−1^/0.34 ng mL^−1^	0.5–10 ng mL^−1^/0.5–30 ng mL^−1^	[65]
	Si@Au/Ir		CLE, GM	Milk, oat, soil, river water	57.23, 84.45 pg mL^−1^/0.89, 1.26 pg mL^−1^	0.0015–100 ng mL^−1^	[87]
	Ti_3_C_2_Tx@Pt		Chloramphenicol (CAP)	Milk, chicken, fish	0.01 µg kg^−1^/0.01 µg kg^−1^	0.0125–0.5 µg kg^−1^/0.0125–1 µg kg^−1^	[88]
	USCGs		CLE	Milk, pork, mutton	0.08 ng mL^−1^/0.03 ng mL^−1^	0.2–2 ng mL^−1^/0.05–1 ng mL^−1^	[89]
	Yeast@IrO_2_		Salbutamol	Pig liver, beef	0.022 ng mL^−1^/0.012 ng mL^−1^	0.02–3 ng mL^−1^/0.045–3 ng mL^−1^	[90]
	APNCs	Colorimetric/catalytic-enhanced colorimetric/photothermal	Enrofloxacin	Milk	0.08 ng mL^−1^/0.01 ng mL^−1^/0.01 ng mL^−1^	0.1–1000 ng mL^−1^/0.01–1000 ng mL^−1^/0.01–1000 ng mL^−1^	[91]
	Ms-Pd/PtNPs		Salbutamol	Pork, beef, animal feed	1 ng mL^−1^/0.01 ng mL^−1^/0.01 ng mL^−1^	-/0.01–100 ng mL^−1^/0.01–100 ng mL^−1^	[92]
	Ru-PDA NPs	Colorimetric/catalytic-enhanced colorimetric/photothermal/fluorescent	Xylazine	Raw milk, orange juice beverage, beer, carbonated beverage, human urine	0.032 ng mL^−1^/0.014 ng mL^−1^/0.0087 ng mL^−1^/0.0041 ng mL^−1^	0.0–1.5 ng mL^−1^/0.05–3 ng mL^−1^/0.01–3 ng mL^−1^/0.01–5 ng mL^−1^	[93]
	CoSAN	Colorimetric/catalytic-enhanced colorimetric/chemiluminescent	Tetracycline (TC)	Milk, honey	0.091 ng mL^−1^/0.062 ng mL^−1^/0.056 ng mL ^−1^	0.1–25 ng mL^−1^/0.1–60 ng mL^−1^/0.1–80 ng mL^−1^	[94]
	CoFe PBAs/WS_2_	Chemiluminescent/photothermal	GM	Milk, urine, serum	0.33 pg mL^−1^/16.67 pg mL^−1^	0.001–100 pg mL^−1^/0.05–100 pg mL^−1^	[95]
Mycotoxins	Au@PtCu TNPs	Colorimetric/catalytic-enhanced colorimetric	Zearalenone (ZEN)	Corn	0.14 ng mL^−1^/0.13 ng mL^−1^	0.14–2.375 ng mL^−1^/0.13–15.5 ng mL^−1^	[96]
	CuCo@PDA		AFB1	Peanut, maize, wheat	8.5 pg mL^−1^/2.2 pg mL^−1^	0.01–50 ng mL^−1^/0.01–500 ng mL^−1^	[97]
	CuS@Au-Pt		Bongkrekic acid (BA)	Tremella, corn flour, millet flour	0.66 ng mL^−1^/1.05 ng mL^−1^	0–50 ng mL^−1^/0–100 ng mL^−1^	[98]
	ESi-AuPt		Fumonisin B1 (FB1), AFB1, ZEN	Rice, corn, lake water	0.0077, 0.032, 0.016 ng mL^−1^/0.061, 0.0016, 0.0054 ng mL^−1^	0.013–1.2, 0.004–0.4, 0.013–0.4 ng mL^−1^/0.013–100, 0.004–100, 0.013–3.7 ng mL^−1^	[99]
	Fe-N-C SAzyme		AFB1, FB1	Maize	44.5, 157 ng mL^−1^/2.8, 13.9 ng mL^−1^	0.005–10, 0.02–10 ng mL^−1^/0.005–200, 0.02–150 ng mL^−1^	[100]
	MnO_2_ NSs		AFB1	Maize	0.109 ng mL^−1^/0.015 ng mL^−1^	0.01–2.5 ng mL^−1^/0.01–150 ng mL^−1^	[59]
	Pt@AuNF		ZEN	Corn	0.065 ng mL^−1^/0.052 ng mL^−1^	0.065–1.418 ng mL^−1^/0.052–7.28 ng mL^−1^	[81]
	ReS_2_@Pt		Deoxynivalenol (DON)	Rice, wheat	0.018 ng mL^−1^/6.5 pg mL^−1^	0.05–12 ng mL^−1^/0.05–30 ng mL^−1^	[101]
	MNPs	Colorimetric/catalytic/magnetic enrichment plus catalysis	AFB1	Rice, corn, peanut	0.34 μg L^−1^/0.17 μg L^−1^/0.023 μg L^−1^	1–6 μg L^−1^/0.1–2 μg L^−1^/0.02–0.4 μg L^−1^	[58]
	AuPt NFs	Colorimetric/catalytic-enhanced colorimetric/photothermal	AFB1	Tap water, semen cassia, peanut, corn	0.05 ng mL^−1^/0.01 ng mL^−1^/0.04 ng mL^−1^	0.005–1 ng mL^−1^/0.01–1 ng mL^−1^/0.05–1 ng mL^−1^	[102]
	Fe_3_O_4_@PDA@Pt		ZEN	Corn grits, corn kernels, rice, rice flour, oat, millet, rye, sorghum, wheat, barley	0.1 μg L^−1^/0.004 μg L^−1^/0.013 μg L^−1^	-/0.009–1.538 μg L^−1^/0.023–3.435 μg L^−1^	[103]
	CPP	Colorimetric/catalytic-enhanced colorimetric/photothermal/fluorescent	DON	Millet, maize	0.032 ng mL^−1^/0.021 ng mL^−1^/0.056 ng mL^−1^/0.5 ng mL^−1^	0.8–40 ng mL^−1^/0.05–30 ng mL^−1^/0.8–30 ng mL^−1^/0.5–1.2 ng mL^−1^	[104]
	ZrFe-MOF@Pt	Colorimetric/catalytic-enhanced colorimetric/fluorescent	Aflatoxin M1	Milk, milk powder	0.0636 ng mL^−1^/0.0179 ng mL^−1^/0.0062 ng mL^−1^	0.0963–0.3971 ng mL^−1^/0.0562–0.8046 ng mL^−1^/0.03906–2.5 ng mL^−1^	[69]
Pesticides	Au@Pt	Colorimetric/catalytic-enhanced colorimetric	ACE	Vegetables	0.33 ng mL^−1^/0.068 ng mL^−1^	5–200 ng mL^−1^/1–150 ng mL^−1^	[105]
	Fe_3_O_4_-MOF-Pt		Carbofuran (CAR)	Chinese cabbage, cucumber, oilseed rape	0.5 ng mL^−1^/0.15 ng mL^−1^	0.25–1.25 ng mL^−1^/0.25–5 ng mL^−1^	[106]
	GO-Pt_30_-AuI		Imidacloprid	Corn, lettuce, lake water, river water	24.66 pg mL^−1^/7.86 pg mL^−1^	-/0.781–50 ng mL^−1^	[62]
	PtPdRu NFs		Thiamethoxam	Cowpea	0.13 ng mL^−1^/0.03 ng mL^−1^	0.27–3.33 ng mL^−1^/0.87–2.83 ng mL^−1^	[107]
	Au@Pt	Colorimetric/catalytic-enhanced colorimetric/photothermal/fluorescent	ACE	Apple	0.098 ng mL^−1^/0.049 ng mL^−1^/0.008 ng mL^−1^/0.038 ng mL^−1^	0.1–10 ng mL^−1^/0.1–50 ng mL^−1^/0.01–50 ng mL^−1^/0.05–20 ng mL^−1^	[108]
	PtPdCo	Colorimetric/catalytic-enhanced colorimetric/fluorescent	ACE	Celery, papaya	1.7 pg mL^−1^/4.9 pg mL^−1^/11.5 pg mL^−1^	-/0.01–500 ng mL^−1^/0.01–50 ng mL^−1^	[109]
	MnO_2_ NFs	Colorimetric/chemiluminescent	Chlorpyrifos	Astragalus, poria cocos, water	0.1 ng mL^−1^/0.033 ng mL^−1^	-/0.1–50 ng mL^−1^	[110]
	Pt/Ti_3_C_2_Tx	Colorimetric/temperature/pressure	Chlorothalonil (CHL)	Astragalus, honeysuckle	5 ng mL^−1^/0.04 ng mL^−1^/0.09 ng mL^−1^	-/0.05–100 ng mL^−1^/0.1–100 ng mL^−1^	[111]
Other hazards	Au@Pt	Colorimetric/catalytic-enhanced colorimetric	Antipyrine	Herbal tea, surface water	4.59, 7.22 ng mL^−1^/3.03, 2 ng mL^−1^	-/5–45, 4–26 ng mL^−1^	[112]
	Au@Pt		Okadaic acid	Seawater, river water, fish	1.5 ng mL^−1^/0.5 ng mL^−1^	2.6–6.7 ng mL^−1^/0.8–6.8 ng mL^−1^	[113]
	Fe_2_NC		Capsaicin	Edible oils	0.0426 ng mL^−1^/0.0224 ng mL^−1^	0.02–4 ng mL^−1^/0.01–1 ng mL^−1^	[114]
	NiCo_2_O_4_		17β-estradiol (E2)	Pork, beef	0.2 ng mL^−1^/1 ng mL^−1^	0–8 ng mL^−1^/0–30 ng mL^−1^	[115]
	GO-Pt_30_-AuIr		Cd^2+^	Corn, lettuce, lake, river	71.25 pg mL^−1^/7.02 pg mL^−1^	1.526–100 ng mL^−1^	[62]
	Si@Au/Ir		Cd^2+^	Milk, oats, soil, river water	97.6 pg mL^−1^/0.65 pg mL^−1^	0.0015–100 ng mL^−1^	[87]
	MOGP	Colorimetric/catalytic-enhanced colorimetric/photothermal	Tetrodotoxin	Pufferfish meat, fish liver, fish skin, fish balls	1 ng mL^−1^/0.01 ng mL^−1^/0.025 ng mL^−1^	1–500 ng mL^−1^/0.01–100 ng mL^−1^/0.025–250 ng mL^−1^	[116]
	AuPt@Cu-HCF	colorimetric/catalytic-enhanced colorimetric/photothermal/catalytic-enhanced photothermal	Diazepam	Lake water, fish	0.82 ng mL^−1^/12.82 pg mL^−1^/12.26 pg mL^−1^/4.43 pg mL^−1^	0.97–4000 ng mL^−1^/0.12–4000 ng mL^−1^/0.12–4000 ng mL^−1^/0.24–4000 ng mL^−1^	[117]
	Au@PB NPs	Colorimetric/catalytic-enhanced colorimetric/photothermal/SERS	Higenamine (HIG)	Urine, functional beverage (Red Bull)	1.07 ng mL^−1^/0.68 ng mL^−1^/0.71 ng mL^−1^/0.01 ng mL^−1^	2–10 ng mL^−1^/1–12 ng mL^−1^/1–14 ng mL^−1^/0.1–1 ng mL^−1^	[118]

a: The references listed in this table primarily cover studies published from 2019 to 2026. b: No relevant data provided.

## Data Availability

This is a comprehensive review manuscript and all the data is contained within this article.

## Abbreviations

Full name	Abbreviation
3,3,5,5-tetramethylbenzidine	TMB
3,3′-diaminobenzidine	DAB
3-amino-9-ethylcarbazole	AEC
Acetamiprid	ACE
Aflatoxin B1	AFB1
Bongkrekic acid	BA
Carbofuran	CAR
Carbon dots	CDs
Catalase	CAT
Catalytic constant	K_cat_
Chloramphenicol	CAP
Chlorothalonil	CHL
Clenbuterol	CLE
Deoxynivalenol	DON
Fumonisin B1	FB1
Gas chromatography	GC
Gas chromatography–mass spectrometry	GC-MS
Gentamicin	GM
Gold nanoparticles	GNPs
Graphene oxides	GO
Higenamine	HIG
High-performance liquid chromatography	HPLC
High-performance liquid chromatography–mass spectrometry	HPLC-MS
Lateral flow immunoassay	LFIA
Limit of detection	LOD
Maximum reaction velocity	V_max_
Metal organic framework	MOF
Michaelis constant	K_m_
Near-infrared	NIR
Oxidase	OXD
Peroxidase	POD
Ractopamine	RAC
Reactive oxygen species	ROS
Scavenging superoxide anions	•O_2_^−^
Surface-enhanced Raman spectroscopy	SERS
Superoxide dismutase	SOD
Tetracycline	TC
Zearalenone	ZEN
